# Molecular Targeting of Ischemic Stroke: The Promise of Naïve and Engineered Extracellular Vesicles

**DOI:** 10.3390/pharmaceutics16121492

**Published:** 2024-11-21

**Authors:** Jihun Lee, Dongho Geum, Dong-Hyuk Park, Jong-Hoon Kim

**Affiliations:** 1 Laboratory of Stem Cells and Tissue Regeneration, Department of Biotechnology, College of Life Sciences and Biotechnology, Korea University, Seoul 02841, Republic of Korea; jhnajung@naver.com; 2 Department of Medical Science, College of Medicine, Korea University, Seoul 02841, Republic of Korea; geumd@korea.ac.kr; 3 Department of Neurosurgery, Anam Hospital, College of Medicine, Korea University, Seoul 02841, Republic of Korea; doctorns@korea.ac.kr

**Keywords:** ischemic stroke, pathophysiology, extracellular vesicles, extracellular vesicle engineering

## Abstract

Ischemic stroke (IS) remains a leading cause of mortality and long-term disability worldwide, with limited therapeutic options available. Despite the success of early interventions, such as tissue-type plasminogen activator administration and mechanical thrombectomy, many patients continue to experience persistent neurological deficits. The pathophysiology of IS is multifaceted, encompassing excitotoxicity, oxidative and nitrosative stress, inflammation, and blood–brain barrier disruption, all of which contribute to neural cell death, further complicating the treatment of IS. Recently, extracellular vesicles (EVs) secreted naturally by various cell types have emerged as promising therapeutic agents because of their ability to facilitate selective cell-to-cell communication, neuroprotection, and tissue regeneration. Furthermore, engineered EVs, designed to enhance targeted delivery and therapeutic cargo, hold the potential to improve their therapeutic benefits by mitigating neuronal damage and promoting neurogenesis and angiogenesis. This review summarizes the characteristics of EVs, the molecular mechanisms underlying IS pathophysiology, and the emerging role of EVs in IS treatment at the molecular level. This review also explores the recent advancements in EV engineering, including the incorporation of specific proteins, RNAs, or pharmacological agents into EVs to enhance their therapeutic efficacy.

## 1. Introduction

Stroke is a neurological disorder caused by acute focal injury of the central nervous system (CNS) with a vascular etiology [1]. According to the World Health Organization, stroke is the second leading cause of death and the fourth leading cause of disability worldwide [2,3]. Ischemic stroke (IS), which accounts for approximately 87% of all strokes, has a steadily increasing incidence and is responsible for nearly half of all stroke-related mortality [4]. IS is defined by neurological dysfunction resulting from focal cerebral, spinal, or retinal infarction, most commonly due to cardioembolism or atherosclerosis in the aortic arch or cervical arteries [1,5]. In clinical practice, the treatment of IS primarily involves intravenous administration of alteplase, the only tissue-type plasminogen activator (tPA) approved by the USFDA for thrombolysis, mechanical thrombectomy, or a combination of both, as quickly as possible after the onset of ischemic events [5,6,7].

Although advancements in infrastructure and treatment technologies have enabled rapid intervention for IS, more than one-third of patients continue to experience temporary or permanent disabilities, such as motor function impairment or dementia [8,9]. These post-stroke complications are closely correlated with both the severity and the frequency of the stroke [10,11]. The primary mechanism of complications arises from damage to brain parenchymal cells due to nutrient-depleted hypoxia and reperfusion injury [12]. The underlying pathophysiological processes include excitotoxicity, oxidative stress, inflammatory responses, disruption of the blood–brain barrier (BBB), and apoptosis [12,13,14]. Since the 1960s, numerous neuroprotective agents have entered clinical trials, but most candidates failed to demonstrate sufficient efficacy, likely due to a lack of preclinical models that accurately mimic the complex pathology of stroke, particularly in elderly populations [14,15,16]. Furthermore, clinical trials involving cell therapies aimed at tissue replacement or the modulation of inflammation have faced significant challenges, including low therapeutic efficacy and unexpected adverse events [17,18,19,20].

Over the last decade, increasing attention has been directed toward the potential of extracellular vesicles (EVs) as an alternative therapeutic strategy for IS [21]. EVs secreted by nearly all cell types facilitate intercellular communication by delivering their luminal cargos to target cells [22]. Moreover, EVs derived from various cell types have been shown to enhance neurogenesis [23], promote angiogenesis [24], attenuate inflammation [25,26], and reduce oxidative stress [27], all of which are critical for the treatment of IS (Table 1). Additionally, the luminal cargos of EVs from damaged tissues reflect disease progression, allowing the EVs to serve as potential biomarkers in various neurological disorders, including stroke [28,29]. Because they can cross the BBB, and their properties and cargos can be modified relatively easily, EVs have emerged as attractive candidates for therapeutic applications in CNS-related disease [30,31].

In this review, we briefly summarize the general characteristics of EVs, describe the molecular mechanisms underlying the pathophysiology of IS, and discuss the naïve EV cargos that target these mechanisms. We also discuss recent advances in EV engineering that aim to improve the therapeutic efficacy of EVs.

**Table 1:** Recent research on the use of naïve extracellular vesicles (EVs) for treatment of ischemic stroke (IS).

EV Source	Major Cargo Molecules	In Vitro Stroke Model	In Vivo Stroke Model				Major Targeted Molecules/Pathway	Outcome	**Reference**
			Animal Model	Administration Route	Dosage	Time Point of Administration			
Rat BM-MSCs	-	-	Rat tMCAO model (2 h)	Tail vein	100 μg	24 h	-	Enhanced neurite remodeling Enhanced neurogenesis and angiogenesis	[23]
Human iPSC-derived MSCs	-	OGD/R-HUVECs (8 h)	Rat tMCAO model (2 h)	Tail vein	1 × 10^11^ particles	4 h	STAT3	Enhanced angiogenesis Reduced autophagy	[24]
HUVECs	miR-1290	OGD/R-neurons (1.5 h)	Mouse tMCAO model (1 h)	Intracranial (AP: 2.0 mm, ML: 1.7 mm, DV: 1.35 mm)	5 μg	Immediately (0 h)	-	Reduced apoptosis	[25]
Human NSCs	-	Glucose-free H/R model (1.5 h)	-	-	-	-	-	Reduced apoptosis and oxidative stress Enhanced axonal elongation Enhanced angiogenesis	[32]
Rat BM-MSCs	-	OGD/R-microglia (1–5 h)	Rat tMCAO model (1.5 h)	Tail vein	120 μg	2 h	cysLT2R ERK1/2	Mitigated microglia M1 polarization	[33]
Human BM-MSCs	-	-	Mouse tMCAO model (0.5 h)	Tail vein	Released by 2 × 10^6^ MSCs	Immediately (0 h)	-	Reduced apoptosis Reduced peripheral immune cell inflitration	[34]
Astrocytes	miR-34c	OGD/R-N2a cells (-)	Rat tMCAO model (-)	Tail vein	-	-	TLR7 NF-κB/MAPK pathway	Reduced apoptosis and inflammation	[35]
Human ESCs	TGF-β and Smad2 and Smad4	-	Mouse tMCAO model (1 h)	Tail vein	1 × 10^9^ particles	2 h and day 1, 2 (3 times)	TGF-β/Smad pathway	Reduced apoptosis and inflammation Reduced peripheral immune cell inflitration	[36]
UC-MSCs	circBBS2	H/R model of SH-SY5Y cells (4 h)	Rat tMCAO model (2 h)	Tail vein	50 μg	4 h and day 1, 2 (3 times)	miR-494	Reduced ferroptosis by upregulation of SLC7A11	[37]
Mouse AD-MSCs	miR-760-3p	OGD/R-N2a cells (4 h)	Mouse tMCAO model (1 h)	Intranasal	10 μg	Day 1, 3, 5 (3 times)	CHAC1	Reduced ferroptosis	[38]
Rat BM-MSCs	-	OGD/R-BV2 and PC12 cells (6 h)	Rat tMCAO model (2 h)	Tail vein	80 μg (low) 100 μg (medium) 120 μg (high)	2 h	-	Shift of microglial polarization state toward M2 phenotype Reduced pyroptosis and inflammation	[39]
Mouse AD-MSCs	miR-25-3p	OGD/R-neurons (10 h)	Mouse tMCAO model (1 h)	Femoral vein	10 μg	Immediately (0 h) or 12 h	p53-BNIP3 signaling	Reduced autophagy	[40]
Human BM-MSCs	-	-	Mouse tMCAO model (0.5 h)	Femoral vein	EVs released by 2 × 10^6^ MSCs	Day 1, 3, 5 (3 times)	-	Neuroprotection Enhanced neurogenesis and angiogenesis Modulated peripheral immune response	[41]

## 2. General Characteristics of EVs

EVs are non-replicative, membrane-bound particles enclosed by a lipid bilayer that are naturally released by cells [42]. EVs are generally categorized into three main types based on their size and biogenesis: exosomes (40–200 nm), microvesicles (200–1000 nm), and apoptotic bodies (500–2000 nm) [43]. Exosomes are formed by the inward budding of the endosomal membrane, leading to the generation of intraluminal vesicles. The intraluminal vesicles are released into the extracellular space through the fusion of multivesicular bodies, also known as late endosomes, with the plasma membrane [44]. Microvesicles and apoptotic bodies are produced and released by the outward budding of the plasma membrane in normal and apoptotic cells, respectively [45]. As different types of EVs often share surface markers and biogenesis mechanisms, the International Society for Extracellular Vesicles recommends using the term “EV” as a general nomenclature for vesicles released from cells, classifying them into small and large EVs based on their size [46].

EV biogenesis involves several key pathways, one of which requires the endosomal sorting complexes required for transport (ESCRT) protein complex. This complex plays a crucial role in cargo recruitment and sorting during EV formation [47]. EVs can also be produced by ESCRT-independent mechanisms, such as the ceramide-based sphingomyelinase (SMase) pathway or the tetraspanin-dependent pathway [48,49]. Tetraspanins (e.g., CD63, CD81, and CD9), ESCRT-associated proteins (e.g., Alix, TSG101, and Syntenin), and heat shock protein 70 (HSP70) are incorporated into EVs during biogenesis and serve as conventional markers for identifying EVs [42]. EVs are also capable of encapsulating a variety of bioactive molecules, including proteins, lipids, and nucleic acids (double-stranded DNAs, mRNAs, and microRNAs [miRNAs]), which they subsequently deliver to target cells [50]. In the CNS, EVs secreted by stimulated cortical neurons are preferentially taken up by neurons rather than glial cells [51]. Additionally, glioblastoma-derived EVs can modulate the local immune environment by transferring the miRNAs miR-451 and miR-21 to monocytes and macrophages in the brain [52].

Once secreted, EVs interact with recipient cells either by being taken up directly or by activating receptors on the plasma membrane [53,54]. The direct uptake of EVs can occur by various mechanisms, including clathrin- or caveolin-mediated endocytosis, phagocytosis, micropinocytosis, lipid raft-mediated endocytosis, and membrane fusion [55]. Specific EV surface proteins, such as MFG-e8 and CD209, play critical roles in facilitating EV uptake by interacting with corresponding surface proteins on recipient cells [56,57]. EV surface proteins can also prevent uptake by nontarget cells; for example, EVs expressing CD47 can evade clearance by monocytes, allowing them to persist and function in an endocrine manner [58].

## 3. The Molecular Pathophysiology of IS and the Therapeutic Potential of EVs

The complications associated with IS arise from pathophysiological changes within the CNS triggered by restricted nutrient and oxygen supply, resulting in the activation of glial cells and the irreversible loss of brain parenchymal cells [12]. Omics data analyses have demonstrated that EVs, particularly those derived from mesenchymal stem cells (MSCs), carry a diverse array of anti-inflammatory or antiapoptotic cargos, while simultaneously promoting neurogenesis and angiogenesis for tissue repair [59]. In this section, we explore the molecular mechanisms driving IS pathologies and discuss the therapeutic potential of naïve EV cargos to mitigate these processes.

### 3.1. Excitotoxicity

**Figure 1:**
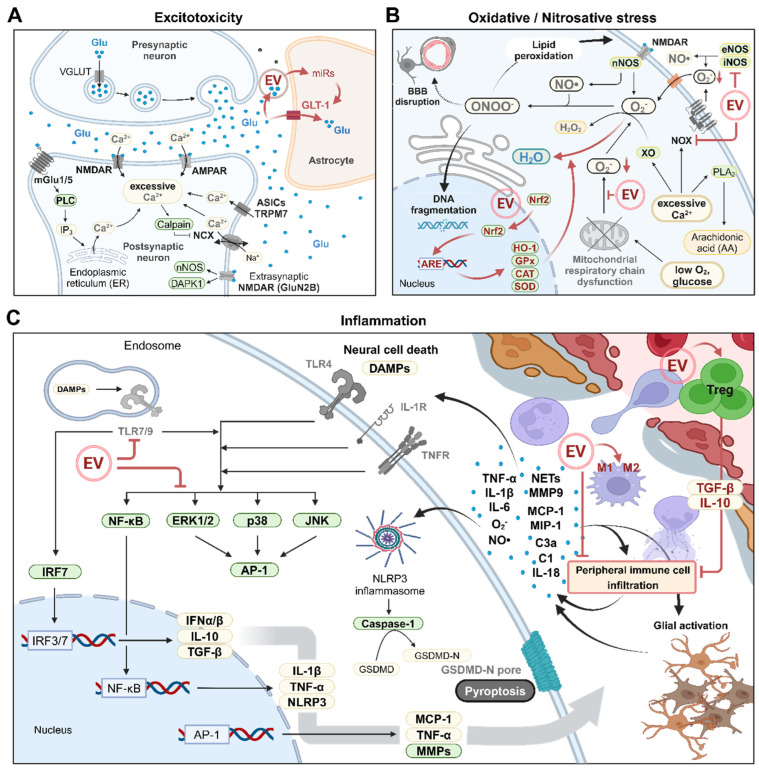
Schematic overview of disease progression and molecular pathophysiology in ischemic stroke. As cerebral blood flow (CBF) decreases, the supply of nutrients and oxygen to the brain tissue is reduced, leading to a decrease in intracellular ATP levels and the formation of reactive oxygen species (ROS). The reduction in ATP levels causes membrane depolarization, resulting in excessive glutamate release at the synapses and triggering excitotoxicity (**A**). Concurrently, mitochondrial ROS formation due to reduced oxygen, along with intracellular calcium overload from excitotoxicity and nNOS activation via N-methyl-D-aspartate receptors (NMDARs), induces oxidative/nitrosative stress. Notably, the peroxynitrite (ONOO-) generated in this process causes severe cellular damage through DNA fragmentation and lipid peroxidation (**B**). Activated astrocytes and microglia secrete proinflammatory cytokines, such as IL-1β and TNF-α, and danger-associated molecular patterns (DAMPs) released from dead cells further trigger inflammation. Oxidative/nitrosative stress and inflammation result in damage to brain endothelial cells, facilitating the infiltration of peripheral immune cells, which exacerbates the pathological process (**C**). The reperfusion of CBF contributes to the worsening pathology via similar mechanisms. Key molecules or pathways targeted by the extracellular vesicles (EV) are highlighted in red color. Green boxes indicate molecules with enzymatic functions. The schematic illustration was created with BioRender.com.

Excitotoxicity refers to nerve cell damage or death caused by excessive stimulation by neurotransmitters. IS begins with a reduction in cerebral blood flow, leading to a decrease in ATP levels that disrupts transmembrane ionic gradients (Figure 1A). This disruption triggers anoxic depolarization and the excessive release of neurotransmitters into the extracellular space [60]. The released neurotransmitters cannot be sufficiently cleared due to the insufficient ATP supply, resulting in their continued accumulation in the extracellular environment. Glutamate, an excitatory neurotransmitter, induces calcium ion (Ca^2+^) influx by activating N-methyl-D-aspartate receptors (NMDARs), kainate receptors, and α-amino-3-hydroxy-5-methyl-4-isoxazole-propionic acid receptors (AMPARs) [61]. The Ca^2+^ overload is further exacerbated by Ca^2+^ release from the endoplasmic reticulum through the mGluR–PLC pathway, calcium-dependent protease (e.g., calpain)-mediated cleavage of the sodium–calcium exchanger (NCX), and the activation of other Ca^2+^-permeable channels, such as acid-sensing ion channels (ASICs) and TRPM7 [62,63,64,65]. Excessive Ca^2+^ accumulation generates free radicals and initiates cell death pathways, ultimately leading to neuronal cell death [66]. Notably, NMDARs containing the GluN2B subunit can directly activate various cell death signals in neurons [66].

The potential use of EVs to treat IS by reducing extracellular glutamate concentrations has not been fully investigated. Recent studies showed that EVs derived from microglia and neurons deliver miR-124 to astrocytes, leading to the upregulation of astrocytic glutamate transporter-1 (GLT-1) expression, which enhances the clearance of extracellular glutamate in normal conditions and in glioma (Figure 1A) [67,68]. Additionally, EVs originating from astrocytes were found to contain glutamate transporters, suggesting a potential role in scavenging extracellular glutamate [69]. Although investigations of therapeutic EVs remain in the early stages, the results so far suggest that EVs might be useful in treating IS by reducing extracellular glutamate levels.

### 3.2. Oxidative/Nitrosative Stress

Oxidative stress in IS typically manifests in three distinct phases [70]. Initially, oxygen and glucose deprivation lead to the decoupling of the mitochondrial respiratory chain, resulting in the accumulation of reduced intermediates and the generation of reactive oxygen species (ROS) (Figure 1B). The second phase involves the production of hydrogen peroxide (H_2_O_2_) as a result of xanthine oxidase (XO) activation. The final phase generally occurs during reoxygenation and is associated with NADPH oxidase (NOX) activation and a subsequent increase in Ca^2+^ concentrations. Concurrently, nitric oxide (NO) production, driven by the activation of the NMDAR (especially those containing the GluN2B subunit)–PSD95–neuronal nitric oxide synthase (nNOS) complex, further contributes to oxidative stress in the third phase [71]. Oxidative stress can also arise during excitotoxicity, primarily by ROS generation due to NOX and phospholipase A2 (PLA2) activation in response to Ca^2+^ influx [72,73]. The ROS and reactive nitrogen species (RNS) generated during IS can directly damage DNA, proteins, membranes, and other cellular compounds, ultimately leading to cell death [74]. For instance, NO and superoxide anions produced by nNOS and NOX activation combine to form highly reactive peroxynitrite, which induces DNA fragmentation, lipid peroxidation, and the disruption of the BBB [75].

Oxidative stress in IS can be mitigated by overexpression and nuclear translocation of the nuclear factor erythroid 2-related factor 2 (Nrf2) transcription factor. In the nucleus, Nrf2 binds to the antioxidant response element (ARE), promoting the expression of antioxidant enzymes, such as heme oxygenase (HO-1), glutathione peroxidase (GPx), catalase (CAT), and superoxide dismutase (SOD) [76,77]. Recent studies showed that neural stem cell (NSC)-derived EVs can stimulate Nrf2 translocation and increase the expression of SOD1, CAT, and GPx-1, thereby reducing intracellular ROS levels in neuronal hypoxia/reperfusion models (Figure 1B) [32]. Additionally, EVs from minipig adipose-derived MSCs (AD-MSCs) were shown to decrease the expression of iNOS, NOX-1, and NOX-2 and reduce oxidized proteins in a rat model of acute IS [78]. Furthermore, human AD-MSC-derived EVs were found to reduce ROS production in H2O2-treated endothelial cells to levels comparable to those in control cultures and restore mitochondrial respiratory chain function by delivering miR-146a-5p [79]. Recent studies demonstrated that EVs from young human donors are enriched with GST or NAMPT and can ameliorate age-related tissue damage by enhancing antioxidant capacity [80,81]. These findings highlight the potential of stem-cell-derived EVs in IS therapy by restoring the antioxidant balance and counteracting oxidative stress.

### 3.3. Inflammation and Ischemia/Reperfusion (I/R) Injury

As IS progresses, excitotoxicity and excessive ROS/RNS generation occur from the center of the infarction (ischemic core). This process escalates with reperfusion, leading to brain cell death and the release of various danger-associated molecular patterns (DAMPs) such as ATP, heat shock protein (HSP), and high mobility group box 1 (HMGB1) [82]. Microglia and astrocytes are subsequently activated by DAMPs, oxidative stress, and other inflammatory signals. Along with other brain cells, such as neurons and endothelial cells, the activated microglia and astrocytes release proinflammatory cytokines (e.g., TNF-α, IL-1β, and IL-6), chemokines (e.g., MCP-1 and MIP-1a), matrix metalloproteinases (MMPs), ROS, and NO, which collectively induce inflammation (Figure 1C) [82,83,84]. These released molecules stimulate the expression of cell adhesion molecules (e.g., ICAM-1 and E/P-selectin) in endothelial cells, initiating the infiltration of peripheral immune cells, including leukocytes, monocytes, and lymphocytes [85]. Neutrophils, the first leukocytes to infiltrate, release large amounts of proinflammatory mediators and neutrophil extracellular traps, which further exacerbate brain injury by intensifying inflammation, releasing ROS/NO, and disrupting the BBB [86]. For instance, MMP-9 contributes to BBB disruption by degrading endothelial cell tight junction proteins (e.g., claudin-5) and cerebrovascular basal lamina proteins (e.g., collagen-4), resulting in detrimental effects including brain edema and hemorrhagic transformation [87]. The complement cascade also plays a role in this process; for example, C3a, an anaphylatoxin, and the C1 protein complex exacerbate I/R injury by promoting leukocyte infiltration and activating endothelial cells [88,89].

The inflammatory reactions in IS are mediated by various signaling pathways. Members of the mitogen-activated protein kinase (MAPK) family, including extracellular signal-regulated kinase 1/2 (ERK1/2), c-Jun N-terminal kinase (JNK), and p38, as well as the NF-κB subunits p65/RelA and p50, are activated in response to external signals, such as DAMPs, ROS, and inflammatory cytokines, leading to the upregulation of proinflammatory mediators that exacerbate IS pathology (Figure 1C) [90,91,92,93,94]. Additionally, Toll-like receptors (TLRs), which serve as upstream regulators of MAPKs and NF-κB, induce inflammation by responding to DAMPs [95].

Recent findings suggest that EVs secreted by various cell types may target inflammatory pathways and offer therapeutic benefits in IS. For example, MSC-derived EVs reduced inflammation in a rodent model of transient middle cerebral artery occlusion (tMCAO), potentially by reversing the CysLT2R-ERK1/2–mediated M1 polarization of microglia or by inhibiting immune cell infiltration from the blood [33,34]. Furthermore, astrocyte-derived EVs carrying miR-34c alleviated I/R injury in in vitro models of oxygen–glucose deprivation/reperfusion and in vivo tMCAO models by downregulating TLR7, NF-κB, and MAPK pathways (Figure 1C) [35]. Anti-inflammatory effects can also be achieved by activating regulatory T cells that secrete anti-inflammatory cytokines, such as IL-10 and TGF-β. Embryonic stem cells also release EVs containing TGF-β, Smad2, and Smad4 and were shown to reduce peripheral immune cell infiltration and neuroinflammation by promoting regulatory T cell expansion [36].

It is important to note that certain molecules and cells that promote inflammation and I/R injury might paradoxically play beneficial roles in IS under specific conditions. For example, ERK1/2 activated by brain-derived neurotrophic factor (BDNF) was shown to inhibit apoptosis by reducing caspase-3 activity in hypoxic–ischemic brain injury [96]. Additionally, MMP-9 and proliferating microglia were shown to contribute to neurovascular remodeling during the later stages of cerebral ischemia [97,98]. Therefore, the therapeutic application of EVs harboring immunomodulatory agents in IS requires the careful consideration of the target signaling molecules and the time window for treatments.

### 3.4. Ischemic Brain Cell Death

**Figure 2:**
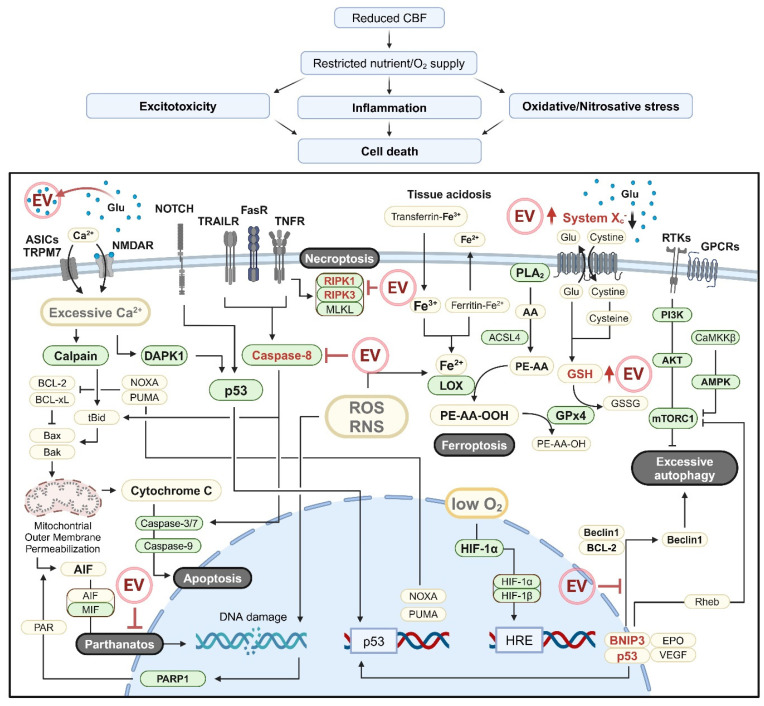
Schematic overview of brain cell death in ischemic stroke. Reduced cerebral blood flow (CBF) leads to decreased nutrient and oxygen supply, triggering excitotoxicity, inflammation, and oxidative/nitrosative stress, which ultimately result in brain cell death. The molecular mechanisms underlying various forms of brain cell death in ischemic stroke are illustrated in the figure. Excitotoxicity-induced receptor activation and intracellular calcium overload contribute to autophagy-related cell death and apoptosis via DAPK1, CaMKKβ, and calpain activation, while also triggering ferroptosis through PLA2 activation, which provides arachidonic acid (AA). Oxidative/nitrosative stress induces mitochondrial outer membrane permeabilization, DNA damage, and lipid peroxidation, driving apoptosis, parthanatos, and ferroptosis. Furthermore, external signals, including proinflammatory cytokines, induce apoptosis and autophagy-related cell death through MAPK and NOTCH signaling, caspase-8 activation, and mTORC1 inhibition. Under the conditions of ATP depletion, rather than activating caspase-8, cell death signaling induced by TNF-α and FasL promotes necroptosis by forming a necrosome complex comprising RIPK1/3 and MLKL. Key molecules or pathways targeted by the extracellular vesicles (EV) are highlighted in red color. Green boxes indicate molecules with enzymatic functions. Schematic illustration was created with BioRender.com.

Various molecular mechanisms contribute to ischemic brain cell death (Figure 2) [99]. One major pathway involves either the activation of the Ca^2+^-dependent protease calpain [100,101] or the induction of the mitochondrial permeability transition due to Ca^2+^ overload and oxidative stress [102,103]. These processes activate the intrinsic apoptosis pathway by causing the release of cytochrome C or apoptosis-inducing factor (AIF) from mitochondria. Additionally, signaling molecules, such as death-associated protein kinase 1 (DAPK1), JNKs, p38, and Notch, which are activated during IS, can induce p53 activation [104]. Once activated, p53 promotes the transcription of proapoptotic genes, such as PUMA and NOXA, which can directly interact with Bcl-xL to permeabilize the outer mitochondrial membrane, ultimately triggering mitochondrial apoptosis [105,106,107].

Extracellular factors released during inflammatory responses, including TNF-α [108], Fas ligand (FasL) [109,110], and TNF-related apoptosis-inducing ligand (TRAIL) [110,111], can induce caspase-8 activation through ligand–receptor interactions, initiating both the extrinsic and the mitochondrial apoptosis pathways (Figure 2) [112,113]. However, caspase activity may also be reduced under IS conditions [114,115]. In such cases, external signals, particularly TNF-α, prompt the formation of the necrosome complex, which consists of receptor-interacting protein kinase (RIPK) 1, RIPK3, and mixed lineage kinase domain-like pseudokinase (MLKL), and induces necroptosis [115,116,117].

Under the acidic conditions of the ischemic brain, the intracellular free iron level increases as a result of ferritinophagy and iron dissociation from transferrin [118,119]. The accumulation of free iron, combined with ROS, enhances lipoxygenase-mediated lipid peroxidation, resulting in the formation of lipid hydroperoxides that trigger ferroptosis [120]. Additionally, excessive intracellular Ca^2+^ activates cytosolic PLA2, promoting the production of arachidonic acid (AA) (Figure 2) [121]. The AA is then esterified into phosphatidylethanolamines by acyl-CoA synthetase long-chain family member 4 (ACSL4), further contributing to ferroptosis [122]. Conversely, GPx-4 utilizes glutathione (GSH) as a substrate to reduce lipid hydroperoxides, thereby inhibiting an iron-dependent cell death ferroptosis [123]. Excessive extracellular glutamate in IS inhibits the cystine/glutamate antiporter (System Xc-), impairing the uptake of cystine, an essential precursor of GSH, thereby reducing GPx-4 activity and increasing susceptibility to ferroptosis (Figure 2) [124]. Moreover, DNA damage caused by excitotoxicity and ROS/RNS activates poly (ADP-ribose) polymerase 1 (PARP1), initiating a series of events, including the cytoplasmic translocation of poly (ADP-ribose), AIF release from mitochondria, AIF/macrophage migration inhibitory factor (MIF) translocation, and DNA cleavage, ultimately resulting in the induction of a cell death pathway known as parthanatos (Figure 2) [125]. Cell death can also be mediated by the inflammasome, a multiprotein complex composed of a single type of sensor protein, such as NLRP or AIM2, along with the adaptor ASC and pro-caspase-1. This complex activates caspase-1 and eventually induces pyroptosis, a type of inflammatory cell death [126,127]. Alternatively, the exposure of phosphatidylserine (PS) on the outer leaflet of the plasma membrane and the expression of proteins that mediate PS recognition (e.g., MerTK and MFG-e8) can trigger phagoptosis, the cell death induced by phagocytosis [128].

Although controversial findings exist, excessive autophagy can exacerbate IS by promoting neuron death [129]. In IS, the inhibition of the phosphoinositide 3-kinase (PI3K)/protein kinase B (AKT) pathway [130] and the activation of ERK1/2 [131] both trigger autophagy-related cell death by inhibiting mammalian target of rapamycin complex 1 (mTORC1) activation (Figure 2). Additionally, the activation of AMP-activated protein kinase (AMPK), either due to a high AMP/ATP ratio [132,133] or by calmodulin-dependent protein kinase kinase β (CaMKKβ) [134], causes autophagy-induced cell death by inhibiting mTORC1. Hypoxia-inducible factor 1 (HIF-1) is also activated in response to hypoxia, leading to the upregulation of p53 and Bcl-2/adenovirus E1B 19-kDa-interacting protein 3 (BNIP3) [135,136], which contributes to excessive autophagy by promoting the release of beclin-1 from the Bcl-2/beclin-1 complex [137] or by inhibiting mTORC1 activation through its binding to Ras homolog enriched in the brain (Rheb) (Figure 2) [138]. In addition, p53 enhances the expression of the damage-regulated autophagy modulator (DRAM) [139], potentially leading to excessive autophagy. Furthermore, the forkhead box O (FOXO) family proteins, which are regulated by Sirt1, AKT, and the signal inducer and activator of transcription 3 (STAT3), can worsen IS outcomes by increasing the expression of autophagy-related proteins, such as ATG7 [140,141,142,143,144]. This dysregulated autophagy can further exacerbate neuronal impairments in IS.

Recent studies suggest that the various forms of cell death observed in IS can be mitigated by EVs. MSC-derived EVs were shown to alleviate both extrinsic and intrinsic apoptosis [145,146]. Specifically, miR-134 delivered by exosomes inhibited the apoptotic cell death of oligodendrocytes by targeting caspase-8 (Figure 2) [146]. In addition, circBBS2 and miR-760-3p enriched in MSC-EVs targeted miR-494 and glutathione-specific gamma-glutamylcyclotransferase 1, respectively, thereby inhibiting ferroptosis by increasing System Xc- activity and boosting GSH levels (Figure 2) [37,38]. Furthermore, EVs derived from bone marrow MSCs were shown to inhibit pyroptosis by reducing the expression of NLRP3, ASC, gasdermin D, and mature IL-1β [39]. Neuronal EVs containing miR-98 were also reported to inhibit phagoptosis by reducing the expression of the platelet-activating factor receptor (PAFR) [147]. Moreover, EVs from AD-MSCs and induced pluripotent stem-cell-derived MSCs were shown to inhibit autophagy-associated cell death by modulating p53–BNIP3 signaling and STAT3 expression, respectively (Figure 2) [24,40]. Although direct studies in IS models are limited, the inhibition of RIPK1/3 and PARP1 expression by MSC-derived EVs in other injury models suggests that these EVs hold potential for inhibiting necroptosis and parthanatos [148,149,150].

## 4. EV Engineering Methods

Advances in understanding the molecular mechanisms underlying IS pathophysiology have led to the development of strategies to target these mechanisms using bioactive molecules. The low permeability of the BBB poses a significant obstacle to non-invasive drug delivery for IS treatment. However, EVs exhibit a range of therapeutic effects in IS-related pathologies, including their ability to cross the BBB, maintain circulation stability, and protect internal cargo with their lipid bilayer. Despite these advantages, the innate therapeutic cargo of naïve EVs may be insufficient to effectively modulate IS pathology, and their delivery to the ischemic region following systemic administration may require further optimization, particularly for therapeutic applications. To address these challenges, various EV modification strategies have been developed to enable the precise regulation of signaling pathways in specific target cells, thereby enhancing their therapeutic capacity, while minimizing adverse effects (Table 2) [151].

**Figure 3:**
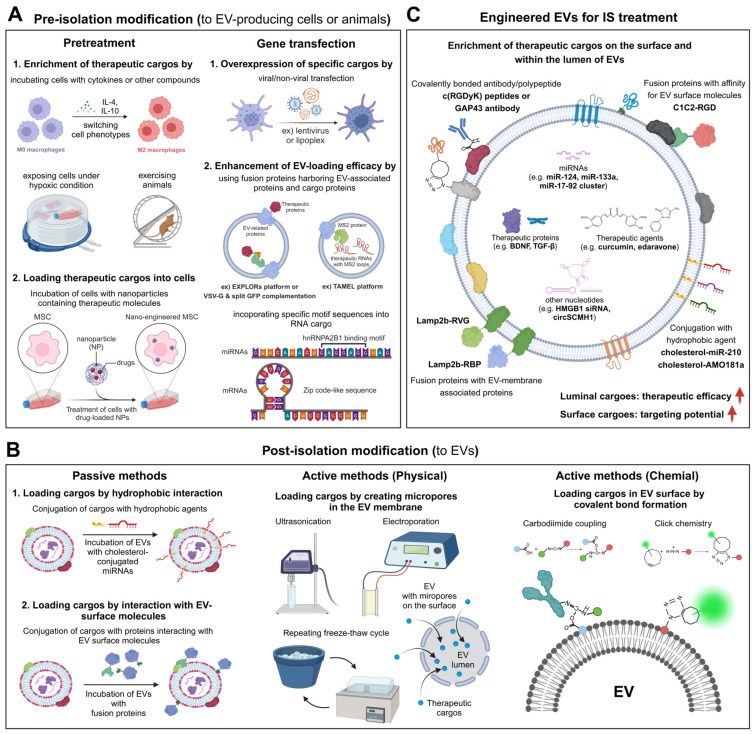
Schematic overview of extracellular vesicle (EV) engineering. Various methods of EV engineering can enhance the therapeutic efficacy of ischemic stroke (IS) treatment. (**A**) Pre-isolation EV engineering involves the modification of the EV-producing cells. Intracellular expression patterns are modulated by altering extracellular conditions or by treating the cells with cytokines, hydrophobic molecules, or molecules capable of cellular uptake, resulting in changes in EV cargos. Desired molecules can be directly loaded into EVs by the transfection of EV-producing cells, the creation of EV-related fusion proteins, or using RNAs containing specific motifs. (**B**) EV engineering is also possible after EV isolation. Hydrophobic molecules or molecules that interact with the EV surface are loaded into EVs by simple co-incubation. Other molecules must be conjugated with hydrophobic agents, such as cholesterol or EV surface-interacting molecules, before co-incubation. Additional post-isolation engineering methods include the microporation of the EV membrane by ultrasonication, electroporation, or freeze–thaw cycles and the use of active loading techniques, such as click chemistry or carbodiimide coupling. (**C**) Different approaches of EV engineering for IS treatment. Schematic illustration was created with BioRender.com.

Generally, EV engineering methods can be categorized depending on whether modifications are made before or after EV isolation (Figure 3). Pre-isolation modifications involve various pretreatments or the gene transfection of EV-producing cells; whereas, post-isolation modifications involve passive methods, such as co-incubation with desired target molecules, as well as active methods that use physicochemical stimulation to enable the loading of exogenous factors [31].

### 4.1. Pre-Isolation Modification: Pretreatment and Gene Transfection

Various pretreatments of EV-producing cells can enhance the efficacy of EVs for IS treatment by altering the composition of the EV cargo. In vitro treatments with cytokines [152,153], metallic compounds [154], magnetic nanoparticles [155], drugs [156], and therapeutic biomolecules [157] under normal or hypoxic culture conditions [158] have been shown to modulate the activity or polarization state of EV-producing cells, including NSCs, MSCs, and macrophages (Figure 3A). These methods either facilitate the loading of extrinsic therapeutic agents into EVs or upregulate the intrinsic levels of neurotrophic, angiogenic, and anti-inflammatory cytokines or cell-survival-related miRNAs within the EVs. These modifications potentiate the ability of EVs to inhibit ischemic brain damage, while promoting tissue regeneration and neurological recovery.

The enrichment of TGF-β, miR-124, and miR-133a in EVs, achieved by hypoxic conditioning or the inflammatory cytokine treatment of EV-producing cells, has been shown to reduce neural cell death in tMCAO animal models [152,153,156]. In addition, the systemic administration of EVs derived from magnetic, nanoparticle-treated MSCs significantly improved the efficiency of EV delivery to IS lesions (5.1 times compared to that without a magnetic field) in a rat MCAO model [155]. Furthermore, exosomes from melatonin-treated rat plasma inhibited microglial and neuronal pyroptosis, partially by downregulating the TLR4/NF-κB pathway [159]. Additionally, mice that underwent moderate exercise before MCAO surgery exhibited increased levels of miR-126 in EVs obtained from circulating endothelial progenitor cells, which enhanced neurogenesis and angiogenesis by promoting BDNF secretion and PI3K/AKT signaling activation [160]. Exercise also elevated miR-484 levels in skeletal-muscle-derived EVs, which targeted ACSL4 and, thereby, inhibited neuronal ferroptosis in a rat model of I/R injury [161].

The gene transfection of EV-producing cells is another commonly used method to modify the contents of EVs. Overexpression to increase the cytosolic levels of therapeutic proteins or RNAs in EV-producing cells can lead to the enrichment of these molecules within EVs (Figure 3A) [162,163,164,165]. Alternatively, specific proteins can be selectively loaded into EVs by creating EV-related fusion proteins [176], using the protein loading platform known as the exosomes for protein loading via optically reversible protein–protein interaction (EXPLORs) [177] or employing VSV-G and split GFP complementation [178]. In addition, specific RNAs can be loaded into EVs by incorporating specific motif sequences (e.g., hnRNPA2B1 binding motif [179] and Zip code-like sequence [180]) in the RNAs or by using the Targeted and Modular EV Loading (TAMEL) platform [181].

### 4.2. Post-Isolation Modification: Passive and Active Methods

One of the simplest methods for engineering EVs is to incubate isolated EVs with molecules that enhance the therapeutic potential of the EVs. Molecules conjugated to hydrophobic lipid derivatives, such as cholesterol and phospholipid–polyethylene glycol, can integrate into the EV membrane during simple incubations (Figure 3B) [166,182]. Non-hydrophobic molecules that have an affinity for EV surface molecules can also be attached to the periphery of EVs by incubation. Additionally, physical stimulation allows for the incorporation of non-hydrophobic molecules in the luminal space of EVs by creating micropores in the EV membrane. Sonication or electroporation of a mixture of EVs and therapeutic molecules is the most common method for actively loading desired cargos into EVs via micropores. Repeated freeze–thaw cycles provide another physical way to actively load molecules into EVs. Cargos can also be loaded into EVs by extrusion, dialysis, or permeabilization with surfactants (e.g., saponin, Triton X-100, or Tween-80) [167,183,184,185]. Recent studies demonstrated that saponin-mediated cargo loading achieved an 11-fold higher efficiency for loading hydrophilic porphyrins compared with passive loading [184]. Furthermore, EVs that were actively loaded with catalase using saponin achieved higher neuron survival rates and more effective ROS removal compared with passively loaded EVs [183].

The surfaces of EVs can be chemically modified by carbodiimide coupling or copper-catalyzed azide-alkyne cycloaddition (click chemistry). Compounds with carbodiimide functional groups, such as 1-ethyl-3-(3-dimethylaminopropyl) carbodiimide hydrochloride, act as crosslinkers by catalyzing linkages between carboxyl and amino groups. Using this chemical method, EV surfaces can be conjugated with antibodies that bind to specific molecules highly expressed in target cells or tissues under pathological conditions (Figure 3B). Additionally, carbodiimide chemistry was used to introduce alkyne groups onto EV surface proteins, which were then conjugated with azide-fluor 545 by click chemistry for the fluorescent labeling of the EVs [186].

### 4.3. EV Engineering for IS Treatment

The therapeutic potential of EVs in IS can be enhanced by modifying the luminal or surface cargo molecules in EVs before or after their isolation using several approaches (Figure 3C). Recent studies demonstrated that increasing the levels of therapeutic proteins, mRNAs, miRNAs, and circRNAs within EVs can enhance the therapeutic efficacy of the EVs for treating IS. For example, the induction of BDNF overexpression in MSCs by lentiviral transfection led to BDNF enrichment in MSC-derived EVs, which reduced apoptosis and inflammation and promoted behavioral recovery and neural repair by activating BDNF–tropomyosin receptor kinase B (TrkB) signaling (Figure 3C) [162]. Furthermore, EVs derived from MSCs induced to overexpress the miR-17-92 cluster by electroporation and lentiviral infection were shown to increase neuronal plasticity, promote neurogenesis and oligodendrogenesis, and improve behavioral outcomes. This effect was achieved by remodeling the cortico-spinal tract and enhancing neuronal innervation via PTEN downregulation and PI3K/AKT/mTOR signaling activation [163,164]. In another study, EVs derived from circSCMH1-overexpressing HEK293T cells enhanced neuronal plasticity, reduced glial activation, and decreased peripheral immune cell infiltration in a mouse model of IS. These therapeutic benefits were mediated by direct binding between circSCMH1 and methyl CpG binding protein 2 (MeCP2), which inhibited the nuclear localization of MeCP2 [165]. Notably, circSCMH1-containing EVs also facilitated functional recovery in a nonhuman primate model of stroke [165].

Pre-incubation of MSC-derived EVs with cholesterol-conjugated miR-210 promoted vascular endothelial growth factor (VEGF) expression and angiogenesis after administration into a mouse MCAO model [166]. Additionally, bone marrow stromal-cell-derived EVs loaded with the hydrophobic anti-inflammatory agent curcumin significantly reduced proinflammatory cytokine secretion and apoptosis after intravenous administration in a mouse model of IS, achieving levels comparable to those observed in normal control mice [168]. Strong free radical scavengers, such as quercetin and baicalin, which were incorporated into EVs by creating micropores using sonication, enhanced the innate potential of the EVs to inhibit ROS generation through the Nrf2/heme oxygenase pathway [167,169]. Additionally, EVs loaded with miR-124 or HMGB1 siRNA by electroporation enhanced neurogenesis and reduced TNF-α expression and apoptosis, respectively [170,171]. Furthermore, EVs that encapsulated the antioxidant drug edaravone by sonication improved the safety and bioavailability of the drug, thereby intensifying its neuroprotective effect [172]. Mouse embryonic stem-cell-derived EVs enriched with curcumin via two or three rapid freeze–thaw cycles enhanced their ability to reduce inflammation, glial activation, and the loss of vascular integrity in a mouse model of IS [173].

The expression of specific peptides on the EV surface can enhance the targeted uptake of EVs by ischemic brain lesions. For instance, the ischemic brain can be targeted by fusing the Lamp2b EV membrane protein with the rabies viral glycoprotein (RVG) peptide, which specifically binds to acetylcholine receptors (Figure 3C) [165]. Lamp2b can also be fused with a peptide (RBP) that binds to the receptor for advanced glycation end products (RAGE), which is highly expressed in the hypoxic cells of the ischemic brain, providing the additional benefit of potentially alleviating DAMP-induced inflammation by directly blocking RAGE [174]. Additionally, a fusion protein consisting of the PS-binding domain (C1C2) of MFG-e8 and the Arg-Gly-Asp (RGD)-4C peptide (ACDCRGDCFC) was attached to EVs by incubation, facilitating the targeted delivery of the EVs to ischemic brain with an approximately 2.5-fold increase in targeting efficiency compared to naïve EVs [175]. During incubation, the C1C2-RGD fusion protein bound to PS, which is abundant on the EV surface. After systemic injection, the interaction between the RGD peptide on EVs and integrin αvβ3, which is highly expressed on reactive cerebral vascular endothelial cells in brain ischemia, enhanced the EV targeting efficiency. Sustained EV delivery was also achieved by directly mixing embryonic, NSC-derived EVs with a glucose/ROS dual-responsive hydrogel [187]. The transplantation of this mixture into the cortex of infarcted brain hemispheres enhanced angiogenesis (approximately two-fold over nontreated IS mice) and improved neurobehavioral recovery. Carbodiimide coupling can be used to conjugate EV surface proteins with an antibody targeting growth-associated protein-43 (GAP43), which shows increased neuronal expression in a rat model of IS (Figure 3C) [167]. This modification effectively enhanced the targeted delivery of the EVs to ischemic brain tissues. Furthermore, EVs treated with dibenzocyclooctyne-sulfo-N-hydroxysuccinimidyl ester (DBCO-sulfo-NHS) can be reacted with the azide-containing cyclo(Arg-Gly-Asp-D-Tyr-Lys) peptide [c(RGDyK)], which has a high affinity for integrin α_v_β_3_ [168]. This copper-free click chemistry method covalently attached c(RGDyK) to the EV surface, thereby facilitating the targeted delivery of EVs to the lesion area in the ischemic brain.

## 5. Discussion

EVs have emerged as important messengers of intercellular crosstalk and gained attention for their crucial roles in diverse physiological and pathological processes across many organs. Numerous studies have explored the application of EVs for the treatment of various diseases. EV-based therapies offer several advantages over traditional cell- or drug-based therapies. A key benefit is that EV therapy significantly reduces concerns related to low cell viability and the risk of thromboembolism associated with intravenous cell injection [188]. Additionally, recent studies have demonstrated that administrating EVs in IS can achieve therapeutic outcomes comparable to those of direct cell injections [41]. Compared to small molecules, EVs exhibit a superior ability to cross the BBB and provide enhanced biocompatibility, with lower cytotoxicity and immunogenicity, as well as fewer adverse effects [188,189]. Engineered EVs, in particular, have shown improved efficiency in targeting ischemic brain lesions, resulting in reduced inflammation and cell death and enhanced neurogenesis and angiogenesis compared with naïve EVs, leading to safer and more effective functional recovery.

Oxidative stress is recognized as a central factor triggering multiple signaling pathways that drive cellular senescence, inflammation, and various forms of neural cell death following IS onset [82,99,190]. Thus, we propose that targeting ROS generation with stem-cell-derived EVs represents an attractive therapeutic approach. In the adult brain, NSCs often acquire senescence phenotypes after ischemic injury, releasing senescence-associated secretory phenotype (SASP) factors that exacerbate neurodegeneration [191,192]. Therefore, rejuvenating NSCs or their niches may serve as a therapeutic intervention to promote brain regeneration. As discussed, EVs derived from stem cells demonstrate the capacity to reduce ROS levels, suggesting their beneficial role in mitigating oxidative stress by reversing the senescent phenotype of NSCs in the ischemic brain. Previous studies have shown that EVs from young animals reduce senescence-associated tissue damage by enhancing antioxidant defense mechanisms [80,81]. In addition, EVs derived from pluripotent stem cells (PSCs) contain proteins and miRNAs involved in anti-senescence and rejuvenation [79,193,194,195]. Our unpublished data also support these findings, showing that stem-cell-derived EVs are enriched with antioxidant proteins and can reverse senescent features of tissue-resident stem cells by boosting GSH levels. Therefore, recent findings from our group and others suggest that stem-cell-derived EVs hold significant potential as senotherapeutics for targeting ROS generation in adult NSCs, thereby promoting brain regeneration after IS.

Another promising application of EVs is the rapid diagnosis of IS. Alteplase, the only drug approved by the USFDA for IS treatment, is a recombinant tPA that is most effective when administered early, ideally within 4–5 h of IS onset, highlighting the importance of rapid diagnosis [6]. EVs derived from blood or cerebrospinal fluid of patients with IS or animal models show altered levels of noncoding RNAs and proteins, offering potential diagnostic markers [196]. For example, circulating EVs from patients with IS and tMCAO rat models exhibit increased levels of miR-20b-5p and miR-93-5p [197]. Inflammatory proteins, such as C-reactive protein (CRP), are also elevated in EVs derived from the serum of patients with acute IS [198]. Furthermore, 67 miRNAs in blood-circulating EVs were found to differ significantly between the ischemic and hemorrhagic subtypes of stroke [199]. This suggests that EVs may help diagnose disease progression and distinguish between major stroke subtypes, enabling more rapid and effective treatments.

The limited blood flow in ischemic regions can be restored by tPA-induced clot lysis. However, tPA may also promote neutrophil degranulation and MMP-9 release, potentially increasing the risk of hemorrhagic transformation [200]. Therefore, several clinical studies are currently investigating EVs as promising alternative treatments for IS (NCT06138210, NCT03384433, and NCT05326724) [201]. A phase 1 clinical trial (NCT06138210) conducted by Xuanwu Hospital is assessing the safety and preliminary efficacy of the intravenous administration of exosomes derived from human-induced pluripotent stem cells (GD-iExo-003) in patients with acute IS. Another phase 1/2 trial (NCT03384433) by Isfahan University of Medical Sciences is investigating the therapeutic effects of allogenic MSC-derived exosomes transfected with miR-124 in patients with acute IS. A pilot study involving five participants with IS who received allogenic placental MSC-derived exosomes showed no adverse effects [202].

The therapeutic application of EVs is still in early stages, and several challenges must be addressed before EVs can be used as therapeutic agents for IS [188,203]. One critical challenge is the reproducible production and quality control of EVs. The characteristics and purity of EVs can vary depending on their source, the environment of the EV-producing cells, and the techniques used for EV isolation and storage. Therefore, it is essential to establish precise and strictly monitored conditions for EV harvesting, along with the use of appropriate markers and analytical methods for quality control. Additionally, a detailed understanding of the in vivo biological activity of administered EVs is needed. Because of the diversity of EV cargos, EV application can affect not only the ischemic brain environment but also the overall physiological conditions of the body. Furthermore, different modes of action may be required depending on whether IS is in the acute or chronic phase. Therefore, therapeutic parameters including administration routes, biodistribution profiles, comprehensive biological functions of EV cargos, optimal treatment timing and dosages, and cytotoxicity profiles must be thoroughly studied to advance the application of EVs as therapeutic agents for IS.

While substantial studies have demonstrated the therapeutic roles of EVs, limited research has focused on the distinct characteristics and biogenesis of EV subpopulations. This limitation is partly due to the challenge of obtaining highly purified EV subtypes in sufficient quantities. Given that the cargo spectrum within EVs may vary across different subtypes, more sophisticated characterization is necessary to fully understand their therapeutic effects in the treatment of IS. Thus, the isolation and classification of EVs remain critical and active areas of discussion and investigation, not only for advancing basic research, but also for their therapeutic applications.

## Figures and Tables

**Table 2:** Recent research in the use of engineered extracellular vesicles (EVs) for treatment of ischemic stroke (IS).

EV Source	Modification	Major Cargo Molecules	In Vitro Stroke Model	In Vivo Stroke Model				Major Targeted Molecules/Pathway	**Outcome**	**Reference**
				Animal Model	Administration Route	Dosage	Time Point of Administration			
BV2 cells	IL-4 pretreatment	miR-124	OGD/R-neurons (45 min)	Mouse tMCAO model (1 h)	Tail vein	100 μg	0 h and day 1, 2 (3 times)	USP14	Reduced apoptosis	[152]
Human NSCs	INF-γ pretreatment	miR-206 and miR-133a-3p and miR-3656	-	Rat tMCAO model (-)	Intracranially (striatum)	4 × 10^9^ particles	24 h	-	Reduced apoptosis and oxidative stress	[153]
Mouse BM-MSCs	Lithium pretreatment	miR-1906	OGD/R-neurons (1 h) OGD/R-microglia (8 h) OGD/R-astrocytes (12 h)	Mouse tMCAO model (1 h)	Femoral vein (day 1) Retro-orbital vein (day 3, 5)	13.5 μg	Day 1, 3, 5 (3 times)	TLR4/NF-κB pathway	Reduced apoptosis Enhanced neurogenesis and angiogenesis Reduced peripheral immune cell inflitration	[154]
Human BM-MSCs	Iron oxide nanoparticle (IONP) pretreatment	IONP and various growth factors	LPS-treated hypoxia-PC12 or rBMDM cells (24 h)	Rat tMCAO model (1 h)	Tail vein	200 μg	Immediately (0 h)	-	Enhanced neurogenesis and angiogenesis Reduced apoptosis and inflammation Shift of macrophage polarization state toward M2 phenotype	[155]
RAW264.7 cells	Edaravone pretreatment	Edaravone	-	Rat pMCAO model	Tail vein	-	Days 1–7 (7 times)	-	Neuroprotection Shift of microglial polarization state toward M2 phenotype	[156]
RAW264.7 cells	Curcumin pretreatment	Curcumin	-	Rat tMCAO model (2 h)	Tail vein	-	Immediately (0 h)	-	Reduced oxidative stress and apoptosis Neuroprotection Attenuated BBB damage	[157]
mouse microglia	OGD/R preconditioning	TGF-β	OGD/R-neurons (6 h) OGD/R-microglia (4 h) OGD/R-bEnd.3 (16 h)	Mouse tMCAO model (1 h)	Femoral vein	10 μg	0 h, 6 h (2 times)	TGF-β/Smad2/3 pathway	Promotion of endothelial cell survival and migration Reduced neuronal apoptosis Enhanced angiogenesis shift of microglial polarization state toward M2 phenotype	[158]
Rat plasma	Melatonin pretreatment	Various miRNAs	-	Rat pMCAO model	Tail vein	100 μg	1 h, 12 h, 36 h (3 times)	NLRP3-mediated pathway and TLR4/NF-κB pathway	Reduced pyroptosis and inflammation	[159]
Circulating endothelial progenitor cells	Treadmill exercise	miR-126	H/R-N2a cells (-)	Mouse pMCAO model	-	-	-	BDNF and PI3k/Akt pathway	Reduced apoptosis Enhanced neurogenesis and angiogenesis	[160]
Rat skeletal muscle	Treadmill exercise	miR-484	OGD/R-PC12 cells (4 h)	Rat tMCAO model (1 h)	Tail vein	-	2 h before operation	ACSL4	Reduced ferroptosis	[161]
Human iPSC-derived MSCs	Transfection (BDNF)	BDNF	-	Mouse tMCAO model (45 min)	Intranasally	1 × 10^10^ particles	2 h, 24 h, 48 h (3 times)	BDNF/TrkB signaling	Reduced apoptosis and inflammation Enhanced neurogenesis and angiogenesis Neuroprotection	[162]
Rat BM-MSCs	Transfection (miR-17-92 cluster)	miR-17-92 cluster	-	Rat tMCAO model (2 h)	Intravenously	100 μg (3 × 10^11^ particles)	24 h	PTEN and PI3k/Akt/mTOR pathway	Enhanced neurite remodeling and neuronal plasticity Enhanced neurogenesis and oligodendrogenesis Enhanced cortico-spinal tract axonal remodeling	[163,164]
HEK293T cells	Transfection (RGV-Lamp2b, circSCMH1)	RGV-Lamp2b and circSCMH1	OGD/R-neurons (3 h)	Mouse photothrombosis (PT) model Mouse dMCAO/tMCAO (1 h) model Rhesus monkey PT stroke model	Mouse: tail vein Rhesus monkey: hind limb vein	Mouse: 12 mg/kg Rhesus monkey: 3 mg/kg	Mouse: 24 h Rhesus monkey: 24 h, 48 h (2 times)	MeCP2	Enhanced neuronal plasticity Reduced glial activation Reduced peripheral immune cell infiltration	[165]
Mouse BM-MSCs	Passive loading (miR-210-cholesterol) Click chemistry (c(RGDyK) peptides)	miR-210 and c(RGDyK) peptides	-	Mouse tMCAO model (0.5 h or 1 h)	Tail vein	100 μg	24 h	VEGF	Enhanced angiogenesis	[166]
Rat blood	Active loading ultrasonication (quercetin) Carbodiimide coupling (GAP43 antibody)	Quercetin and GAP43 antibody	OGD/R-SH-SY5Y cells (1 h)	Rat tMCAO model (2 h)	Tail vein	-	24 h	GAP43 and Nrf2/HO-1 pathway	Reduced apoptosis and oxidative stress	[167]
Mouse BM-MSCs	Passive loading (curcumin) Click chemistry (c(RGDyK) peptides)	Curcumin and c(RGDyK) peptides	-	Mouse tMCAO model (1 h)	Tail vein	300 μg	12 h	NF-κB	Reduced apoptosis and inflammation	[168]
RAW264.7 cells	Active loading ultrasonication (baicalin)	Baicalin	OGD/R-SH-SY5Y cells (1 h)	Rat pMCAO/tMCAO (2 h) model	Tail vein	1.6 mg baicalin	Immediately (0 h)	Nrf2/HO-1 pathway	Reduced apoptosis and oxidative stress	[169]
Mouse BM-MSCs	Transfection (RVG-Lamp2b) Active loading electroporation (miR-124)	miR-124	-	Mouse PT stroke model	Tail vein	-	Day 1	Gli3 and Stat3	Enhanced neurogenesis	[170]
HEK293T cells	Transfection (RVG-Lamp2b) Active loading electroporation (HMGB1 siRNA)	HMGB1 siRNA	-	Rat tMCAO model (1 h)	Tail vein	30 μg siRNAs	18 h before operation	HMGB1	Reduced apoptosis and inflammation	[171]
Rat plasma	Active loading ultrasonication (edaravone)	Edaravone	-	Rat pMCAO model	Tail vein	10 mg/kg edaravone	Days 1–7 (7 times)	-	Neuroprotection	[172]
Mouse ESCs	Active loading freeze–thawing (curcumin)	Curcumin	-	Mouse tMCAO model (40 min)	Intranasally	-	Days 0–7 (twice a day)	-	Reduced oxidative stress and inflammation Reduced glial activation and loss of vascular integrity	[173]
HEK293T cells	Transfection (RBP-Lamp2b) Passive loading (AMO181a-cholesterol)	RBP-Lamp2b and AMO181a	Hypoxia-Neuro2A cells (24 h)	Rat tMCAO model (1 h)	Intranasally	75 μg	1 h	RAGE and miR-181a	Reduced apoptosis and inflammation	[174]
Human NPCs (ReN cells)	Passive loading (RGD-C1C2)	RGD-C1C2 and various miRNAs	-	Mouse tMCAO model (1 h)	Tail vein	300 μg	12 h	p38 MAPK pathway	Reduced inflammation	[175]

## Data Availability

Details are available from authors.
